# Correction: Firoznezhad et al. Formulation and In Vitro Efficacy Assessment of *Teucrium marum* Extract Loading Hyalurosomes Enriched with Tween 80 and Glycerol. *Nanomaterials* 2022, *12,* 1096

**DOI:** 10.3390/nano15221729

**Published:** 2025-11-17

**Authors:** Mohammad Firoznezhad, Ines Castangia, Carlo Ignazio Giovanni Tuberoso, Filippo Cottiglia, Francesca Marongiu, Marco Porceddu, Iris Usach, Elvira Escribano-Ferrer, Maria Letizia Manca, Maria Manconi

**Affiliations:** 1 Department of Pharmacy, University of Salerno, 84084 Fisciano, Italy; m_firoznejhad_20500@yahoo.com; 2 Department of Scienze della Vita e dell’Ambiente, University of Cagliari, 09124 Cagliari, Italy; tuberoso@unica.it (C.I.G.T.); cottiglf@unica.it (F.C.); fmarongiu@unica.it (F.M.); porceddu.marco@unica.it (M.P.); mlmanca@unica.it (M.L.M.); manconi@unica.it (M.M.); 3 Sardinian Germplasm Bank (BG-SAR), Hortus Botanicus Karalitanus (HBK), University of Cagliari, 09124 Cagliari, Italy; 4 Department of Pharmacy and Pharmaceutical Technology and Parasitology, University of Valencia, Burjassot, 46100 Valencia, Spain; iris.usach@uv.es; 5 Biopharmaceutics and Pharmacokinetics Unit, Institute for Nanoscience and Nanotechnology, University of Barcelona, 08028 Barcelona, Spain; eescribano@ub.edu

## Figure

In the original publication [1], there was a mistake in Figure 6.

An incorrect image was mistakenly used due to the handling of a large number of similar microscopic images during figure preparation. The authors have now replaced the incorrect image with the correct one.

The original publication has also been updated.

The authors apologize for any inconvenience caused by this error.

The corrected Figure 6 should appear as follows:

The authors state that the scientific conclusions are unaffected. This correction was approved by the Academic Editor.

## Figures and Tables

**Figure 6:**
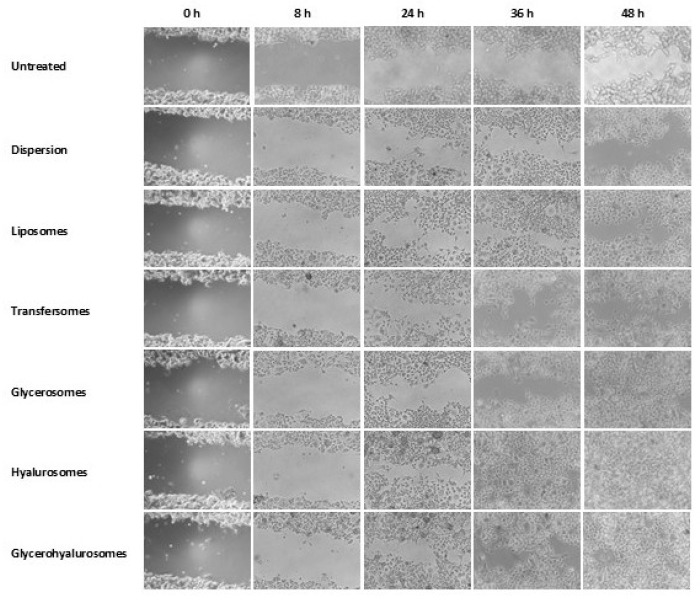
Representative images of the scratch performed in fibroblast monolayers and treated with the extract in dispersion or loaded in vesicles.
